# Identification and Characterization of Type IV Pili as the Cellular Receptor of Broad Host Range *Stenotrophomonas maltophilia* Bacteriophages DLP1 and DLP2

**DOI:** 10.3390/v10060338

**Published:** 2018-06-20

**Authors:** Jaclyn G. McCutcheon, Danielle L. Peters, Jonathan J. Dennis

**Affiliations:** CW405 Biological Sciences Building, 11455 Saskatchewan Dr. NW, Department of Biological Sciences, University of Alberta, Edmonton, AB T6G 2E9, Canada; jgmccutc@ualberta.ca (J.G.M.); dlpeters@ualberta.ca (D.L.P.)

**Keywords:** bacteriophage, phage, phage therapy, phage receptor, *Stenotrophomonas maltophilia*, *Pseudomonas aeruginosa*, Type IV pili, pilus

## Abstract

Bacteriophages DLP1 and DLP2 are capable of infecting both *Stenotrophomonas maltophilia* and *Pseudomonas aeruginosa* strains, two highly antibiotic resistant bacterial pathogens, which is unusual for phages that typically exhibit extremely limited host range. To explain their unusual cross-order infectivity and differences in host range, we have identified the type IV pilus as the primary receptor for attachment. Screening of a *P. aeruginosa* PA01 mutant library, a host that is susceptible to DLP1 but not DLP2, identified DLP1-resistant mutants with disruptions in pilus structural and regulatory components. Subsequent complementation of the disrupted pilin subunit genes in PA01 restored DLP1 infection. Clean deletion of the major pilin subunit, *pilA*, in *S. maltophilia* strains D1585 and 280 prevented phage binding and lysis by both DLP1 and DLP2, and complementation restored infection by both. Transmission electron microscopy shows a clear interaction between DLP1 and pili of both D1585 and PA01. These results support the identity of the type IV pilus as the receptor for DLP1 and DLP2 infection across their broad host ranges. This research further characterizes DLP1 and DLP2 as potential “anti-virulence” phage therapy candidates for the treatment of multidrug resistant bacteria from multiple genera.

## 1. Introduction

The increasing antimicrobial resistance of bacterial infections in recent years is a global health concern, and is predicted to cause 10 million deaths annually worldwide surpassing those caused by cancer by 2050 [1], creating the possibility of a “post-antibiotic era” in the 21st century. One concern is the emerging pathogenic bacterium *Stenotrophomonas maltophilia*, which is rapidly increasing in prevalence in nosocomial and community-acquired infections [2,3,4]. This bacterium is ubiquitous in the environment and is easily transmitted between immuno-compromised patients and health care providers through direct contact and cough-generated aerosols [2,4]. Most commonly associated with respiratory infections, *S. maltophilia* can also cause severe bacteremia, meningitis, endocarditis, pneumonia and catheter-related bacteremia/septicemia. Once a *S. maltophilia* infection is established, treatment is difficult due to its innate resistance to a broad range of antibiotics including trimethoprim-sulfamethoxazole, β-lactams, macrolides, cephalosporins, fluoroquinolones, aminoglycosides, carbapenems, chloramphenicol, tetracyclines, and polymyxins [2,4]. *S. maltophilia* can also be found in polymicrobial infections with the opportunistic, multidrug resistant pathogen *Pseudomonas aeruginosa* [4]. *P. aeruginosa* is a major cause of nosocomial infections, particularly in immuno-compromised individuals such as AIDS, burn and cancer patients, and is the most prevalent pathogen found in the lungs of adult cystic fibrosis patients [5,6]. The inability to treat or control *S. maltophilia* and *P. aeruginosa* infections due to their intrinsic and adaptive multi-drug resistance and range of virulence factors, including type IV pili and biofilm formation [7], increases mortality and morbidity and exemplifies the need for alternative treatments to combat these antibiotic resistant bacteria.

The clinical application of bacteriophages to selectively kill target bacteria, known as phage therapy, shows promise as an alternative treatment of antibiotic resistant bacterial pathogens. Discovered a century ago, phages are ubiquitous viruses that exclusively attack and lyse specific target bacteria through adsorption to the host cell surface, multiplying exponentially as they kill bacteria [6]. Recent studies utilizing phage therapy in animal models [4,5,6,8,9,10] and human clinical trials [11,12,13] show the successful eradication of multidrug resistant bacterial infections by specific phage and demonstrate that phage therapy can be a successful treatment option in humans with no apparent side effects [14,15]. The specificity of phages for their hosts relies upon the presence of the correct surface receptor. Whereas spontaneous mutation of phage’s cell surface receptors allows a bacterium to become resistant to phage infection, it can also attenuate the bacterium’s fitness or pathogenicity if the phage targets a virulence factor, in what is termed an “anti-virulence strategy”. Identifying bacterial receptors for phages such as the type IV pilus or lipopolysaccharide [16], and characterizing the mechanisms of phage-host interaction will enhance the development of phage cocktails targeting different receptors and creating evolutionary pressure towards bacterial avirulence.

While bacteriophages typically have a very narrow host range, recently discovered novel phages such as DLP1 and DLP2 are capable of infecting across taxonomic orders, using strains from both *S. maltophilia* and *P. aeruginosa* as hosts for propagation [17]. This unique ability suggests that DLP1 and DLP2 may be good candidate phages for use in phage therapy, as their unusually broad host ranges would minimize the number of different phages needed in one treatment. Extensive characterization of bacteriophages is essential before they can be used in clinical trials, for example, to ensure that they do not increase bacterial virulence through lysogenic conversion [18,19]. Therefore, the objective of the experiments presented in this paper is to understand the modes of infection of DLP1 and DLP2 across their broad host ranges. Herein, we identify the type IV pilus as the receptor for DLP1 and DLP2 infection of both *S. maltophilia* and *P. aeruginosa* strains. Identification of the mechanism of attachment for both phages has implications for their use as therapeutic agents and suggests that evolutionarily conserved bacterial specialized surface structures have been adopted by some phages as primary receptors for initial host cell interactions.

## 2. Materials and Methods

### 2.1. Bacterial Strains, Phage and Growth Conditions

Bacterial strains, bacteriophages and plasmids used in this study are listed in Table 1. The *S. maltophilia* strain D1585 was acquired from the Canadian *Burkholderia cepacia* complex Research and Referral Repository (Vancouver, BC) and *S. maltophilia* strain 280 was gifted from The Provincial Laboratory for Public Health—North (Microbiology), Alberta Health Services. The mini-Tn5-*luxCDABE P. aeruginosa* PA01 mutant library used for the receptor screen was a kind gift from S. Lewenza [20]. Additional PA01 mutants were obtained from the University of Washington *P. aeruginosa* transposon mutant library constructed with either an IS*phoA*/hah or IS*lacZ*/hah Tn5 IS50L derivative transposon [21,22]. *P. aeruginosa* and *S. maltophilia* strains were grown aerobically overnight at 30 °C on half-strength Luria Bertani (½ LB) solid medium or in ½ LB broth with shaking at 225 rpm, and *Escherichia coli* strains were grown at 37 °C in full LB, unless otherwise noted. Media was supplemented with antibiotics at the following final concentrations when necessary (μg per mL): gentamicin (Gm), 10 for *E. coli* and 35 for *P. aeruginosa*; chloramphenicol (Cm), 35 for *E. coli* and *S. maltophilia* D1585 and 75 for *S. maltophilia* 280; and tetracycline (Tc), 10 for *E. coli*, 50 for 280, and 100 for D1585.

Bacteriophages DLP1 and DLP2 were previously isolated on *S. maltophilia* strain D1585 and partially characterized, with the results subsequently reported [17]. DLP1 and DLP2 belong to the phage family *Siphoviridae* and are both capable of infecting across taxonomic orders, lysing different strains of *S. maltophilia* in addition to *P. aeruginosa.* Bacteriophage φKZ belongs to the phage family *Myoviridae* and infects *P. aeruginosa* strains [23]. Propagation of DLP1, DLP2, and φKZ were performed using soft agar overlays as previously described [17]. Briefly, 100 μL of culture was incubated with 100 μL of phage for 20 min, mixed with 3 mL of 0.7% ½ LB top agar, and overlaid onto plates of ½ LB solid media [24]. Plates were incubated at 30 °C overnight until plaques formed. Plates with confluent lysis were used to make high titer stocks by overlaying with 3 mL of modified suspension medium (SM) (50 mM Tris-HCl pH 7.5, 100 mM NaCl, 10 mM MgSO_4_), collecting the top agar and incubating for 30 min at room temperature on a platform rocker with 20 μL chloroform per plate. The supernatant was collected after centrifugation for 5 min at 10,000× *g* and filter sterilized using a Millex-HA 0.45 μm syringe-driven filter unit (Millipore, Billerica, MA, USA) and stored at 4 °C. Titer of stocks was obtained using serial dilutions of phage stock into SM in the soft agar overlay technique with *S. maltophilia* D1585 for DLP1 and DLP2, and *P. aeruginosa* PA01 for φKZ.

### 2.2. Transposon Mutant Library Receptor Screen

A 2242 member *P. aeruginosa* PA01 random-insertion mini-Tn5-*luxCDABE* transposon mutant library [20] was screened for resistance to DLP1 phage infection using a spotting assay. 100 μL overnight culture was spread on ½ LB solid medium and allowed to dry. 10 μL of DLP1 was spotted in duplicate, as well as 10 μL of phage φKZ and ½ LB as positive and negative controls, respectively. Plates were incubated overnight at 30 °C and examined for absence of DLP1 clearing the following day. High titer phage stocks of 10^10^ pfu/mL were used.

### 2.3. Phage Plaquing Assays

DLP1 and DLP2 plaquing ability was determined by spotting on bacterial soft agar overlays. Briefly, 100 μL of overnight culture was mixed with 3 mL of 0.7% ½ LB top agar, overlaid onto ½ LB plates with or without antibiotics and allowed to dry at room temperature for 30 min. Phage stocks were standardized to 10^10^ PFU/mL on *S. maltophilia* D1585 and tenfold serially diluted in SM to 10^3^ PFU/mL. 5 μL of each dilution was spotted onto the prepared plates and incubated for 18 h at 30 °C. Each experiment was repeated in biological and technical triplicate.

### 2.4. Construction of ΔpilA S. maltophilia D1585 and 280 Mutants

The major pilin subunit, *pilA*, was identified in *S. maltophilia* D1585 by sequence homology to *pilA* in *P. aeruginosa* PA01 using Geneious (10.1.3) [32], and was subsequently used to identify the *pilA* ortholog in *S. maltophilia* 280. The amino acid sequence percent identity of pilin subunits were compared using MUSCLE [33,34]. The *S. maltophilia* D1585 and 280 clean deletion *pilA* mutants were constructed by allelic exchange [35] as described below, using primers listed in Supplementary Table S1.

Two separate PCRs were performed to amplify DNA fragments 1096 bp and 955 bp in length, corresponding to regions upstream and downstream of the *pilA* gene in D1585, respectively, with 30 nucleotides of overlap at the 3′ and 5′ ends. Primers were designed from a 6361 bp contig containing the *pilA* gene, as the D1585 genome assembly is currently incomplete. The sequence upstream to the region to be deleted was amplified from D1585 genomic DNA using primers Sm*pilA*upF and Sm*pilA*upR-OE. The sequence downstream of the deletion was amplified from D1585 genomic DNA using primers Sm*pilA*downF-OE and Sm*pilA*downR. Primers to delete *pilA* in *S. maltophilia* 280 were designed similarly from an 111,798 bp contig containing *pilA*, as the 280-genome assembly is also incomplete. The region upstream of the deletion was amplified from 280 genomic DNA using primers 280*pilA*upR and 280*pilA*upF-OE, producing a 1074 bp product. The downstream region was amplified using primers 280*pilA*downR-OE and 280*pilA*downF, producing a 1,146 bp product. The PCR mixture contained 50 ng D1585 genomic DNA, 0.5 μM of each primer, 0.2 mM dNTPs, 3% DMSO and 1 × GC Buffer (New England Biolabs, Mississauga, ON, Canada) in sterile milliQ water and was heated for 3 min at 98 °C before the addition of 1 U of Phusion High-Fidelity DNA Polymerase (New England Biolabs) per reaction. The reactions were then processed for 35 cycles of 15 s at 98 °C, 30 s at 57.4 °C for D1585 or 66.7 °C for 280, and 30 s at 72 °C before a final extension of 10 min at 72 °C. The PCR products were purified using a QIAquick PCR purification kit (Qiagen, Inc., Germantown, MD, USA).

Overlap extension PCR [36] was used to join the upstream and downstream PCR products, creating a 2021 bp template for D1585 and a 2190 bp template for 280. Briefly, a 1:1 ratio of upstream and downstream template was added to a PCR mixture lacking primers and processed for 3 min at 98 °C, during which time Phusion polymerase was added, followed by 35 cycles of 15 s at 98 °C, 30 s at 67.8 °C for D1585 and 65.3 °C for 280, and 1 min at 72 °C before a final extension of 10 min at 72 °C. A 1:1 ratio of primers Sm*pilA*upF and Sm*pilA*downR or 280*pilA*upR and 280*pilA*downF was added to the reaction after 10 cycles, which allowed the upstream and downstream templates to prime off their 30 bp overlap. The ~2 kb products were purified from a 1% agarose gel using a Gene Clean II kit (MP Biomedicals, Santa Ana, CA, USA) and digested with *Sal*I and *Hind*III Fast Digest restriction endonucleases (Thermo Scientific, Waltham, MA, USA). The fragments were cloned into pEX18Tc, yielding pD1585Δ*pilA* containing a 444 bp in-frame deletion within the 477 bp D1585 *pilA* gene and p280Δ*pilA* containing a 372 bp in-frame deletion within the 414 bp 280 *pilA* gene as confirmed by Sanger sequencing. The deletion vectors were transformed into the mobilizing *E. coli* strain S17-1 and the plasmids were transferred into D1585 or 280 by conjugation as described previously, in a 1:10 donor to recipient ratio [37]. Single crossover D1585 transconjugants carrying pD1585Δ*pilA* in their chromosome were selected on LB agar containing 100 μg/mL tetracycline and merodiploid status was verified by PCR using *pilA* specific primers, Sm*pilA*F and Sm*pilA*R lacking restriction enzyme tails. Single crossover 280 transconjugants carrying p280Δ*pilA* were selected on LB agar containing 50 μg/mL tetracycline and merodiploid status was verified by PCR using *pilA* specific primers, 280*pilA*F and 280*pilA*R lacking restriction enzyme tails. Positive transconjugants were grown in the absence of tetracycline for 2 h to allow for a second crossover and screened on LB agar containing 10% (*w*/*v*) sucrose. Sucrose-resistant colonies appearing after 48 h incubation at 37 °C were screened for the presence of the *pilA* deletion using the *pilA* specific primer pairs.

### 2.5. Complementation of Pilus Mutants

The *pilA* and *pilE* genes were amplified from *P. aeruginosa* PA01 by colony PCR using primer pairs Pa*pilA*F and Pa*pilA*R, and Pa*pilE*F and Pa*pilE*R respectively, and from *S. maltophilia* D1585 genomic DNA by PCR using primer pairs Sm*pilA*F and Sm*pilA*R, and Sm*pilE*F and Sm*pilE*R, as listed in Supplementary Table S1. The *pilA* gene was amplified from *S. maltophilia* 280 genomic DNA by PCR using primer pairs 280*pilA*F and 280*pilA*R. The resulting products were digested with *Sal*I and *Hind*III, or *Bam*HI and *Hind*III Fast Digest restriction endonucleases (Thermo Scientific) and ligated using T4 DNA ligase (NEB) into the vector pUCP22 [30] for expression in PA01, or pBBR1MCS [29] for expression in D1585 and 280. The resulting constructs as listed in Table 1 were verified by Sanger sequencing and subcloned into electrocompetent *E. coli* DH5α before transforming *P. aeruginosa* PA01 and *S. maltophilia* D1585 and 280 mutants by electroporation.

Electrocompetent *P. aeruginosa* PA01 cells were prepared as described by Choi et al. (2006) [38] with some modifications. Briefly, overnight cultures of PA01 grown in LB at 37 °C were harvested by centrifugation for 5 min at 8000× *g* and were washed 3 times with 300 mM sucrose. The cell pellet was resuspended in the remaining 300 mM sucrose and competent cells were stored in 100 μL aliquots at −80 °C prior to use. Electrocompetent *S. maltophilia* D1585 and 280 cells were prepared as described by Ye et al. (2014) [39]. Overnight cultures were subcultured and grown to an optical density at 600 nm (OD_600_) of 1.0 in LB at 37 °C and placed on ice for 30 min. The chilled cells were harvested by centrifugation for 5 min at 4000× *g* and 4 °C and washed 3 times with ice-cold 10% glycerol (*v*/*v*). The competent cells were resuspended in residual 10% glycerol and stored in 100 μL aliquots at −80 °C prior to use. Electrocompetent *E. coli* DH5α cells were prepared similarly to *S. maltophilia*; however, subcultures were grown to an OD_600_ of 0.5–0.7 at 37 °C.

### 2.6. Transmission Electron Microscopy

Bacterial samples were prepared for electron microscopy as follows. Overnight cultures were diluted 1:20 in fresh ½ LB broth and grown to an OD_600_ of 0.3–0.6 at 30 °C with shaking. 1 mL of subculture was harvested at 15,000× g, fixed in EM fixative (2.5% glutaraldehyde, 2% paraformaldehyde, 0.1 M phosphate buffer, pH 7.2) for 30 min, and resuspended in 1× phosphate-buffered saline (PBS), pH 7.4. For visualization of bacteria, a carbon-coated copper grid was incubated with 10 μL of sample for 2 min and stained with 2% phosphotungstic acid (PTA) for 10 s. To visualization phage binding, the bacterial samples were mixed in a 1:2 ratio with high titer 10^10^ pfu/mL phage stock for 2 min. 10 μL of this mixture was incubated on the copper grid for 4 min, followed by staining with 2% PTA. Transmission electron micrographs were captured using a Philips/FEI Morgagni transmission electron microscope with charge-coupled device camera at 80 kV (University of Alberta Department of Biological Sciences Advanced Microscopy Facility).

### 2.7. Twitching Motility Assay

Twitching motility assays were used as an indirect measurement of type IV pili function. A single bacterial colony was suspended in 100 μL LB broth and stab inoculated with a toothpick through a 3 mm thick LB agar layer (1% agar), containing 0.3% porcine mucin or antibiotic where indicated, to the bottom of the petri dish and incubated with humidity at 37 °C for 24 h for PA01 [40] or 72 h for D1585 [41]. Twitching motility zones between the agar and petri dish interface were visualized by gently removing the agar and staining each plate with 1% (*w*/*v*) crystal violet for 30 min followed by rinsing excess stain away with water. Stained twitching zone areas were measured using ImageJ software (NIH, Bethesda, MD, USA) [42]. Each strain was tested in biological and technical triplicate and average twitching area was calculated from the nine twitching zones.

### 2.8. Bioinformatic Analysis

Experimentally determined pili-binding *Siphoviridae* phages were identified in a search of the literature and the corresponding genomic sequence was used to perform a conserved domain search (CD-search) [43] to identify the potential presence of a phage-tail_3 domain which is found within gp26 (central tail hub) of DLP1 and DLP2. The CD-search database CDD v3.16–50369 PSSMs was used to identify phage-tail_3 domains above the expected E-value threshold of 0.01. Composition-based statistics adjustment was used. The identified protein featuring the phage-tail_3 domain for each phage was then used for a multiple sequence alignment to include DLP1 and DLP2 using the MUSCLE [33,34] plugin for Geneious [32]. Two multiple sequence alignments were also performed with the top 10 BLASTP hits for gp26 of DLP1 and DLP2. For each MUSCLE alignment, the maximum number of iterations selected was 8, with the anchor optimization option selected. The trees from iterations 1 and 2 were not retained. The distance measure for iteration 1 was kmer6_6 and was pctid_kimura for subsequent iterations. The clustering method was UPGMB for all iterations.

## 3. Results and Discussion

### 3.1. P. aeruginosa PA01 Type IV Pilus Mutants Are Resistant to DLP1 Infection

Bacteriophage DLP1 is a broad host range phage capable of lysing eight out of 27 *S. maltophilia* and two out of 19 *P. aeruginosa* strains tested, one being the reference strain PA01 [17]. A spotting screen of 2242 PA01 mutants with random mini-Tn5-*luxCDABE* transposon insertions causing polar mutations [20] identified 27 mutants (Supplementary Table S2) with insertions in 11 different genes that were resistant to DLP1 infection (Table 2). Ten of the 11 genes disrupted are directly involved in type IV pilus biogenesis, including both structural components, *pilB*, *pilE*, *pilT*, *pilV*, *pilY1*, and *fimV*, and regulatory components, *pilJ*, *pilR*, *pilS*, and *algR*. The additional gene, *PA2806*, encodes a conserved hypothetical protein, with homology to QueF, an NADPH-dependent 7-cyano-7-deazaguanine reductase enzyme involved in queuosine biosynthesis, with unknown function related to pilus biogenesis. While the pilus related genes identified in the mutant library cover only a fraction of the over 40 genes involved in type IV pilus biogenesis and function in *P. aeruginosa* [44], there were no other pilus mutants in the library to screen for DLP1 sensitivity.

To better identify the type IV pilus as the receptor for DLP1 infection of PA01, additional PAO1 pilus mutants were obtained [21,22] and screened. These included transposon mutants of the major pilin subunit PilA, the outer membrane pore subunit PilQ, and additional structural subunits PilF, PilN and PilU (Table 2). As expected, these mutants were also resistant to DLP1 infection; however, the *pilU* mutant was not. Similar results have been observed following infection of mutant *P. aeruginosa* strains PA01 and PAK by another pilus-dependent *Siphoviridae* bacteriophage, P04; the unpiliated *pilB* and hyperpiliated *pilT* mutants are resistant to phage infection, whereas the hyperpiliated *pilU* mutant remains susceptible [45]. These genes encode the three ATPases that are responsible for extension and retraction of the type IV pilus; PilB is involved in polymerization of pilin subunits, and PilT and PilU are involved in depolymerization [46]. Assembly and disassembly of the pilus allows bacteria to move across a surface, a process known as twitching motility. While PilT and PilU appear to have similar functions, only *pilU* mutants have the unusual combination of pilus-specific phage susceptibility and loss of twitching motility [45,46]. Assessment of twitching motility in each of the 27 DLP1 resistant PA01 mutants, as well as the *pilU* mutant, revealed that all lack a twitching zone and therefore functional pili, except for the *PA2806* mutant. These findings mirror what others have observed for pilus-specific phages P04, B3, and D3112 [45], and support the hypothesis that DLP1 uses the type IV pilus for first contact with its host and requires a pilus functionally capable of retraction in order to infect.

### 3.2. Complementation in P. aeruginosa Restores DLP1 Infectivity

**Figure 1:**
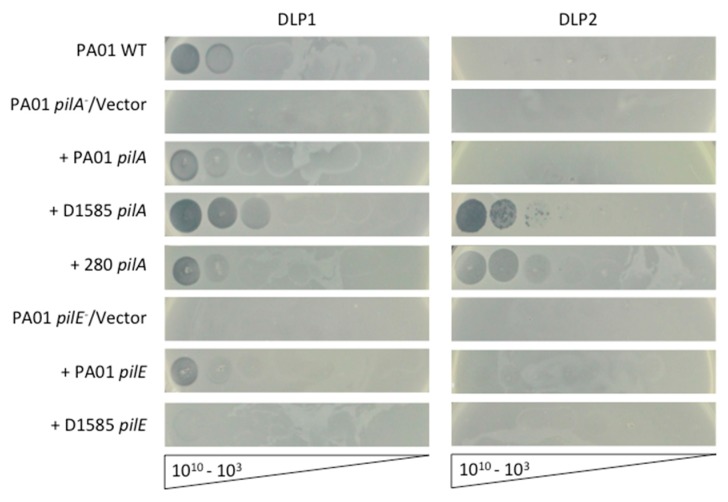
Infection of *P. aeruginosa* PA01 expressing varying pilin subunits by DLP1 and DLP2. PA01 wildtype (WT) is susceptible to DLP1, while the PA01 *pilA* PW8621 and *pilE* PA01_lux_41_C7 mutants are resistant to infection. Complementation of PA01 mutants with the endogenous genes restores DLP1 infectivity to wildtype levels, clearing at 10^9^ PFU/mL. Cross-genera complementation with the *S. maltophilia* D1585 *pilA* gene restores infection by DLP1, clearing at 10^8^ PFU/mL, and allows DLP2 plaquing at 10^7^ PFU/mL. Complementation with the D1585 *pilE* gene allows partial DLP1 infection. Cross-genera complementation with the *S. maltophilia* 280 *pilA* gene also allows DLP2 infection at 10^8^ PFU/mL and partially restores DLP1 infectivity. Images are representative of three biological replicates, each with three technical replicates. Similar results were observed for the additional *pilA* and *pilE* mutants when complemented.

**Figure 2:**
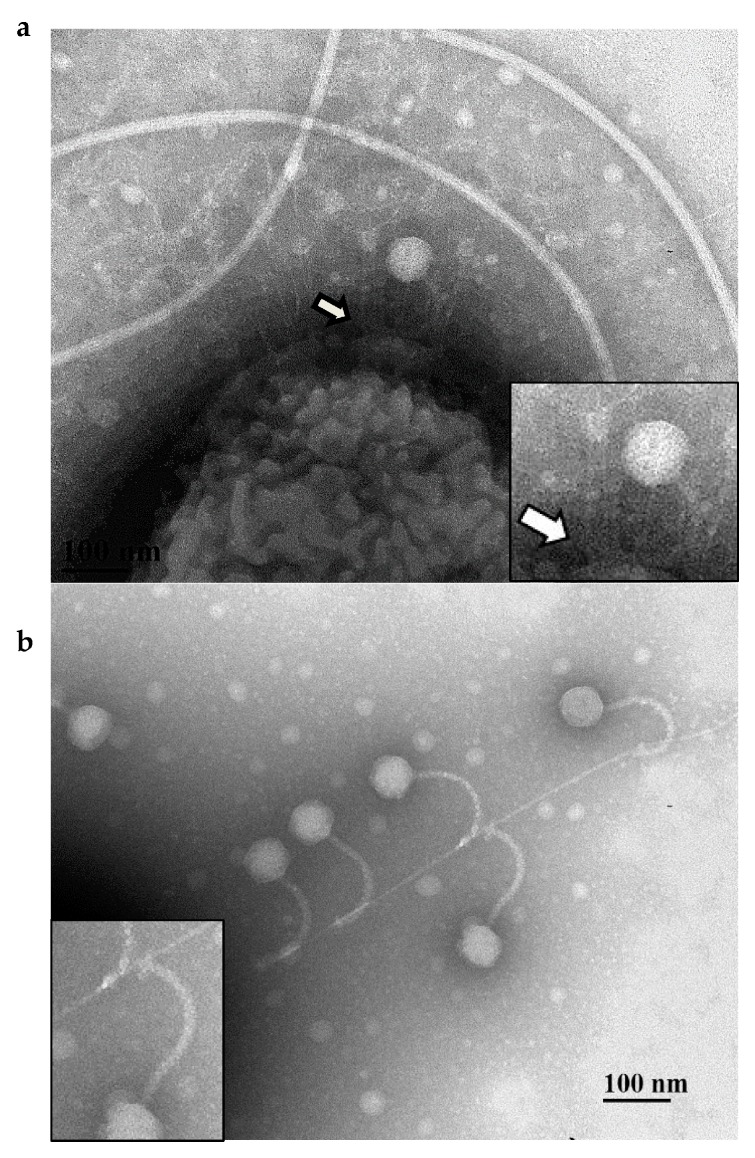
DLP1 interacts with pili on the cell surface of wildtype *S. maltophilia* D1585 and *P. aeruginosa* PA01. Electron micrographs showing (**a**) multiple pili projecting from the pole of a PA01 cell with a single DLP1 phage interacting with the base of a pilus (arrow). Cells and phage were stained with 2% phosphotungstic acid and visualized at 110,000-fold magnification by transmission electron microscopy. (**b**) Five DLP1 phage binding a single pili extending from the pole of a D1585 cell. Inset images show closer view of phage-pili interactions. Cells and phage were at 110,000-fold magnification.

To confirm that PA01 mutants were resistant to DLP1 infection due to their lack of pili, the two major subunit *pilA* and three minor subunit *pilE* mutants were chosen as hosts for complementation analysis and to assess DLP1 infectivity via phage plaquing assays. In PA01, *pilE* is the seventh gene in the minor pilin operon and *pilA* is transcribed as a single gene, therefore polar mutations are not a concern for complementation of these mutants. Wildtype PA01 is susceptible to DLP1 but not DLP2, clearing at 10^9^ PFU/mL (Figure 1). Both *pilA* mutants, PW8621 and PW8622, are resistant to DLP1 infection and when transformed with the endogenous PA01 *pilA* gene, exhibit restored susceptibility to DLP1 infection. DLP1 deposited on bacterial lawns of the complemented *pilA* mutants produce clear spots comparable to wildtype levels. The same effect was observed for each of the three PA01 *pilE* mutants transformed with pUCP22 carrying the endogenous *pilE* gene. In comparison, transformation of each mutant with an empty pUCP22 vector did not restore DLP1 infection and no lysis of the bacterial lawn was observed. As confirmation that DLP1 binds type IV pili expressed on the surface of PA01, transmission electron microscopy (TEM) was used to visualize this interaction. Imaging of log phase PA01 cells mixed with high titer DLP1 showed phage particles near the cell surface that appeared to interact with the base of a pilus via the phage tail (Figure 2a). This observation, along with complementation restoring phage infectivity, confirms the pilus as phage DLP1′s initial point of attachment to *P. aeruginosa* PA01.

**Figure 3:**
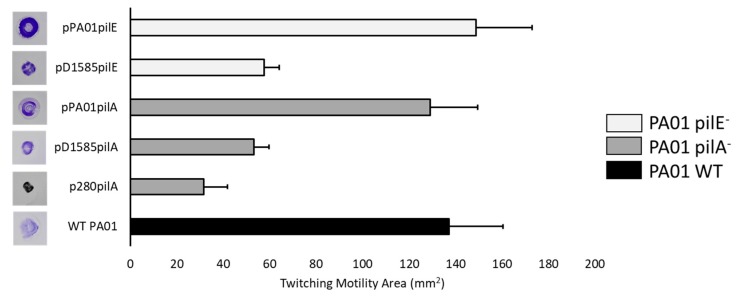
Twitching motility is partially restored in cross-genera complemented *P. aeruginosa* PA01 pilin mutants. PA01, its *pilA* PW8621 and *pilE* PA01_lux_41_C7 mutants and their respective complemented strains were stab inoculated through 1% ½ LB agar and incubated for 24 h at 37 °C. Twitching zones were visualized with 1% crystal violet and measured using ImageJ [42]. Complementation with the endogenous PA01 genes restored twitching to wildtype, while cross-genera complementation only partially restored motility. Representative twitching zones are shown on the left and the average area of the twitching zones from nine replicates are shown on the right including error bars showing standard deviation.

To determine whether DLP1 also uses the type IV pilus as the first point of contact with its *S. maltophilia* hosts, we performed cross-genera complementation experiments using the PA01 minor pilin, *pilE*, and major pilin, *pilA*, orthologs in strain D1585 expressed in the respective PA01 mutant. Both DLP1 and DLP2 were isolated on *S. maltophilia* strain D1585, and out of the 27 strains tested, both phages infect D1585 with equally high efficiency, producing plaques when spotted at 10^3^ PFU/mL [17]. Therefore, we describe D1585 as the major host for DLP1 and DLP2 in our *S. maltophilia* strain collection. Similar to complementation with the endogenous PA01 genes, cross-genera complementation of the PA01 *pilA* mutants PW8621 and PW8622 with D1585 *pilA* also restored DLP1 infection. Exposure of these cross-genera complemented PA01 mutants to DLP1 produced infection at the same efficiency of plating as wildtype PA01; however, DLP1 appears to clear the bacterial lawn expressing D1585 *pilA* more effectively (Figure 1). DLP1 infects *S. maltophilia* D1585 at higher efficiency of plating, plaquing at 10^3^ PFU/mL, as compared to *P. aeruginosa* PA01 that DLP1 is unable to infect at a PFU per mL lower than 10^8^. It is likely that DLP1 binds amino acids in the PilA of D1585 with more affinity than the PilA of PA01. Therefore, expression of the D1585 *pilA* subunit in a *pilA* deficient PA01 strain permits more efficient DLP1 receptor binding and infection, which produces more clear spots in the bacterial lawn. Alternatively, cross-genera complementation of the three PA01 *pilE* mutants with D1585 *pilE* produces only partial infection by DLP1, showing a slightly thinned lawn at 10^10^ PFU/mL (Figure 1). The *pilE* gene encodes one of four minor pilin subunits in *P. aeruginosa* that assemble together at the tip of the pilus, along with FimU and PilY1, to prime pilus assembly [47]. *P. aeruginosa* strains express one of five major type IV pilin alleles with an associated set of minor pilin alleles [48,49]. Studies have shown that the minor pilin genes are compatible with major pilins of the same group, but do not function as well when expressed with a heterologous major pilin [49]. Because the PA01 and D1585 PilA subunits and PilE subunits share only 51% and 43% amino acid sequence identity, respectively, it is possible that the major pilin *pilA* subunits are sufficiently different between *P. aeruginosa* PA01 and *S. maltophilia* D1585 that the D1585 PilE minor subunit does not have high affinity for the PA01 PilA major subunit. This may decrease the association between the minor pilin priming complex and PilA such that the pilus does not assemble proficiently, resulting in decreased piliation or inefficient pilus extension and decreased phage infection, as observed, due to loss of receptor expression. Examination of twitching motility in each of the complemented strains supports this hypothesis, showing that pili function is reduced by approximately 61% and 58% for D1585 *pilA* and *pilE* cross-genera complementation respectively, compared to complementation with the PA01 endogenous subunits (Figure 3). While the overall pili function is similar between D1585 *piE* and *pilA* complemented PA01 mutants, differences in phage infectivity may be explained by changes in amino acids between the foreign and endogenous subunits. This is similar to observations by Giltner et al. 2011; *P. aeruginosa* PA01 Group II *pilE* mutants complemented with a PA14 Group III *pilE* gene in trans decreased twitching motility by 9% relative to endogenous complementation [49]. Because the amino acid sequence identity of PA01 and D1585 PilE subunits is lower than PA01 and PA14 PilE products that share 51% amino acid identity, our substantial decrease in twitching motility is likely due to the inefficient assembly of D1585 PilE with the PA01 pilin subunits.

A second *S. maltophilia* phage, DLP2, was tested against the *pilA* cross-genera complemented strains PW8621 and PW8622 carrying D1585 *pilA* on pUCP22. DLP2 is another broad host range phage that is capable of infecting nine out of 27 *S. maltophilia* strains, including D1585, and two out of 19 *P. aeruginosa* strains, although PA01 is not one of them [17]. Phage spotting shows that DLP2 can infect *pilA* deficient PA01 mutants expressing the D1585 *pilA* gene, clearing the bacterial lawn at 10^9^ PFU/mL (Figure 1). This is not entirely surprising given that DLP2 can infect two different strains of *P. aeruginosa*, HER1004 and 14,715 [17], suggesting that there are no intracellular blocks to phage infection across these genera once a primary receptor for DLP2 is expressed on the cell surface. DLP1 and DLP2 are closely related phages, sharing a high degree of sequence identity over their genomes [17]. Because both are capable of infecting *S. maltophilia* D1585 as a major host, it is possible if not probable that they share the same receptor. These results suggest that DLP2 also uses the type IV pilus as the primary receptor for infection of D1585, requiring only the D1585 major pilin expressed in trans to infect the previously resistant strain, *P. aeruginosa* PA01. However, it is then unclear why the host ranges of DLP1 and DLP2 differ, and how these two phages adhere to pilin subunits of different hosts if they both do adhere to the PilA subunit to infect D1585. Rescue of phage infection through cross-genera complementation of the major pilin subunit also suggests that the pre-pilin signal cleavage sequence of D1585 pilins is conserved and recognized by *P. aeruginosa* pre-pilin peptidase, allowing proficient assembly of mature pilins sufficient for phage recognition and infection.

Based upon the similarity between pilin subunits and the highly conserved nature of type IV pili assembly machinery, heterologous expression of type IV pilins has been used to analyze structure-function relationships of pili in several pathogenic bacteria. Research shows that *P. aeruginosa* can assemble exogenous pilins from species including *Dichelobacter nodosus*, *Moraxella bovis*, *Neisseria gonorrhoeae*, and *Escherichia coli* [50,51,52,53]. Heterologous expression of pili subunits restores pili function and associated phenotypes, such as natural competence and phage binding [54,55,56]. For example, the major pilin subunit PilA from *P. aeruginosa* can be successfully expressed and assembled into functional type IV pili in *N. gonorrhoeae*, and is sufficient for *P. aeruginosa* specific phage PO4 binding, determined through transmission electron microscopy [57]. In contrast to our cross-genera complementation, many of these studies use retraction-deficient *pilT-* strains of *P. aeruginosa* to compensate for low steady-state expression of pili. However, such a technique would inhibit DLP1 and DLP2 infection of the host, as these phages appear to require pili retraction by the host to reach the cell surface. While pilin sequences vary within species, the type IV pilus assembly machinery is widely conserved at the nucleotide level, providing relaxed specificity for the heterologous expression of pilin proteins from distantly related species [49]. This insensitivity to sequence changes in PilA provides an evolutionary benefit to the cell, allowing the incorporation of a wide range of pilins for antigenic variation and functional diversity.

### 3.3. Deletion of pilA in S. maltophilia D1585 Prevents DLP1 and DLP2 Infection

Following on the results obtained from cross-genera complementation that implicated the type IV pilus in D1585 as the receptor for DLP1 and DLP2, the major subunit *pilA* ortholog was deleted in *S. maltophilia* D1585 using overlap-extension PCR and allele exchange to create a clean deletion. Sanger sequencing confirmed the in-frame clean deletion and twitching motility was subsequently examined in both wildtype D1585 and the Δ*pilA* mutant to analyze pili function. D1585 wildtype produces a small zone of twitching, averaging 25 ± 10 mm^2^ after 72 h incubation at 37 °C. This twitching zone is absent in the D1585 Δ*pilA* mutant, indicating that the mutant cannot assemble functional type IV pili, and suggests that the deleted gene encodes the major type IV pilin subunit in D1585. While the sizes of twitching motility zones vary greatly in both clinical and environmental *S. maltophilia* strains [7,41], our D1585 wildtype strain did not consistently produce twitching zones. To further confirm that the D1585 Δ*pilA* mutant was incapable of twitching motility, we induced pili expression in both the wildtype and mutant strains by adding mucin to the media. Mucin is a major component of mucus produced in the lungs where *S. maltophilia* can colonize and has been shown to increase the expression of type IV pili in *P. aeruginosa* resulting in increased twitching motility zones [58]. The addition of 0.3% mucin to the twitching motility plates increased D1585 wildtype twitching zones to approximately 41 ± 14 mm^2^ after only 24 h incubation. This increase in motility, while also inconsistent, was completely absent in the Δ*pilA* mutant, indicating that the mutant does not express functional pili.

**Figure 4:**
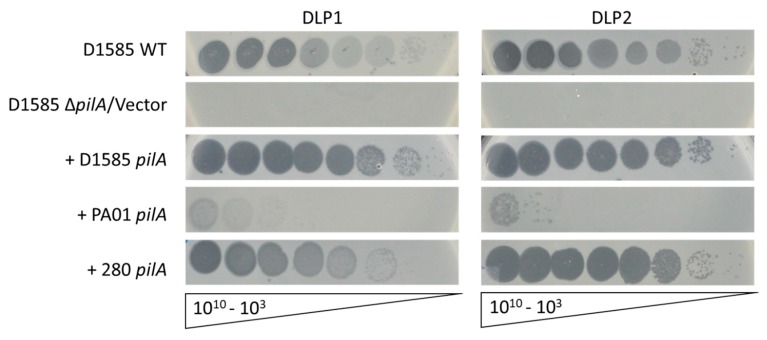
Infection of *S. maltophilia* D1585 expressing varying pilin subunits by DLP1 and DLP2. D1585 wildtype (WT) is susceptible to DLP1 and DLP2, while the D1585 Δ*pilA* mutant is resistant to both phages. Complementation of D1585 Δ*pilA* with the endogenous *pilA* gene restores DLP1 and DLP2 infectivity to wildtype levels, each plaquing at 10^3^ PFU/mL. Cross-genera complementation with the *P. aeruginosa* PA01 *pilA* gene restores partial infection by DLP1 and DLP2, plaquing at 10^7^ and 10^9^ respectively. Cross-species complementation with the *S. maltophilia* 280 *pilA* gene restores DLP2 infection to wildtype levels, and partially restores DLP1 infectivity, showing plaquing at 10^5^. Images are representative of three biological replicates, each with three technical replicates.

Assessment of the phage plaquing ability on the constructed D1585 Δ*pilA* mutant by spot assay shows that the mutant is resistant to infection by DLP1 and DLP2, displaying an absence of clearing and cell lysis at high phage titer (Figure 4). Complementation of the mutant with the endogenous D1585 *pilA* gene restored infection by DLP1 and DLP2 to wildtype levels, each producing plaques at 10^3^ PFU/mL, as expected for type IV pili-specific bacteriophages. Transformation of D1585 Δ*pilA* with an empty pBBR1MCS vector did not restore phage infection and no change in bacterial growth in each phage spot was observed. In contrast to the original characterization of DLP1 by Peters et al. (2015) [17], high titer phage stocks of 10^10^ PFU/mL were able to clear the bacterial lawn and plaque formation was no longer delayed. We suspect that the efficiency of DLP1 infection has increased since its original isolation due to repeated propagation on the *S. maltophilia* host D1585 under laboratory conditions. These results confirm the identification of the type IV pilus as the primary receptor for DLP1 and DLP2 infection of their shared host, D1585. Because DLP1 can infect both D1585 and PA01 via adherence to the type IV pilus, we hypothesized that expression of the exogenous PA01 *pilA* gene in our D1585 Δ*pilA* mutant should restore DLP1 binding and infection, similar to the reverse situation as described above. As expected, cross-genera complementation of the D1585 Δ*pilA* mutant with the cloned PA01 *pilA* gene produced less efficient DLP1 infection, forming plaques when spotted with 10^7^ PFU/mL DLP1 (Figure 4). Surprisingly, DLP2 was also capable of low-level infection of D1585 Δ*pilA* expressing the PA01 major pilin subunit; DLP2 produced plaques at 10^9^ PFU/mL, approximately 10^2^-fold lower efficiency than DLP1. While DLP2 is unable to infect wildtype PA01, it is possible that the PA01 PilA subunit folds differently in D1585 to expose different phage binding sites and enable low levels of DLP2 infection. Alternatively, PA01 PilA may interact with the pilus priming minor pilin subunits of D1585 as efficiently as the endogenous major subunit, perhaps permitting DLP2 to recognize the pili via the minor pilins and reach a surface secondary receptor for partial infection. Twitching motility analysis of the complemented D1585 mutant yielded no changes in motility compared to the low levels observed in wildtype D1585.

TEM visualization of log phase D1585 cells mixed with high titer DLP1 confirmed that DLP1 binds the type IV pilus of D1585. The *S. maltophilia* D1585 viewed expressed multiple pili from their poles; however, the pilus morphology differed from *P. aeruginosa* PA01; D1585 pili were longer and thicker than the fine projections viewed on PA01 (Figure 2). Cells mixed with DLP1 clearly showed phage particles distributed tail first along D1585 type IV pili filaments, with phage appearing to attach to the sides of the pili via the tail fibers, confirming that DLP1′s initial point of attachment to *S. maltophilia* D1585 is the type IV pilus (Figure 2b). In addition to PA01 pili being finer than those of D1585, they were also on average shorter. It is possible that the length of the pili affects the susceptibility of these strains to DLP1 and DLP2. Attempts to visualize DLP2 binding the pili of D1585 were unsuccessful.

### 3.4. Deletion of pilA in S. maltophilia 280 Prevents DLP2 Infection

**Figure 5:**
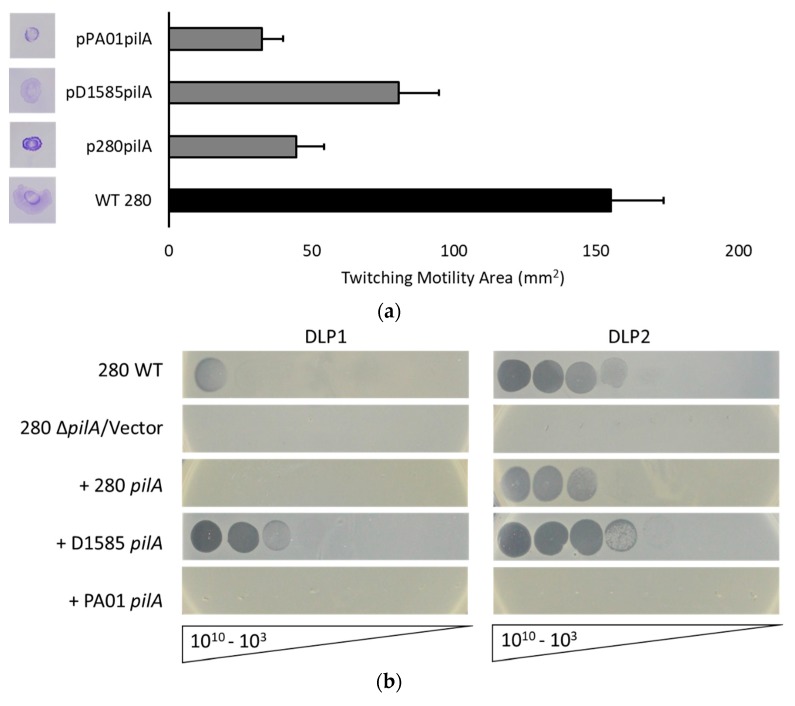
Infection of *S. maltophilia* 280 expressing varying pilin subunits by DLP1 and DLP2. (**a**) Twitching motility of the 280 Δ*pilA* mutant complemented with the PA01, D1585 or endogenous 280 *pilA* is not restored to wildtype levels and is not correlated with phage susceptibility. Representative twitching zones are shown on the left and the average area of the twitching zones from nine replicates are shown on the right. (**b**) 280 wildtype (WT) is susceptible to DLP2, while the 280 Δ*pilA* mutant is resistant. Complementation of 280 Δ*pilA* with the endogenous *pilA* gene restores DLP2 infectivity to near wildtype levels, plaquing at 10^7^ PFU/mL. Cross-species complementation with the *S. maltophilia* D1585 *pilA* gene restores DLP2 infectivity to wildtype levels, plaquing at 10^5^ PFU/mL, and allows partial DLP1 infectivity, showing plaquing at 10^8^ PFU/mL. Cross-genera complementation with the *P. aeruginosa* PA01 *pilA* gene does not restore phage infection. Images are representative of three biological replicates, each with three technical replicates.

As described above, DLP1 and DLP2 infection of *S. maltophilia* D1585 relies on the presence of the type IV pilus for cell surface attachment. To verify that DLP2 uses the type IV pili across its host range and possibly explain differences in the host ranges of DLP1 and DLP2, we examined the *S. maltophilia* strain 280 that is highly susceptible to DLP2 but not DLP1. *S. maltophilia* 280 expresses functional pili, demonstrated by a twitching motility zone approximately 155 mm^2^, 6-fold greater than D1585, following 72 h incubation (Figure 5a). This twitching zone also increased in size when examined on media containing 0.3% mucin, similarly to D1585, increasing to 250 ± 22 mm^2^ after 24 h incubation. Log phase 280 cells viewed by TEM revealed long pili projections from the sides of the cells rather than from the poles. These pili were similar in length and diameter to *S. maltophilia* strain D1585; however, attempts to visualize DLP2 interacting with 280 pili or the cell surface have been unsuccessful due to difficulties in preparing clean samples expressing pili.

A 280 Δ*pilA* mutant was also constructed using overlap extension PCR and allele exchange to delete the D1585 major pilin subunit *pilA* ortholog. Sanger sequencing of the 1 kb regions flanking the deletion confirmed the in-frame clean deletion and assessment of twitching motility on plain ½ LB and ½ LB supplemented with 0.3% mucin revealed the absence of a twitching zone, consistent with a lack of the PilA major pilin subunit and a non-functional type IV pilus. Exposure of the 280 Δ*pilA* mutant to bacteriophage DLP2 via spot assay showed no evidence of cell lysis, indicating that this mutant is resistant to DLP2 infection, similar to D1585 Δ*pilA* (Figure 5b). Complementation of 280 Δ*pilA* with the endogenous *pilA* gene restored DLP2 infection to near wildtype levels, producing plaques at 10^7^ PFU/mL as compared to 10^5^ PFU/mL on wildtype. These results confirm that DLP2 uses the type IV pilus as its cell surface receptor for infection of *S. maltophilia* 280 in addition to strain D1585.

Similar to cross-genera complementation of the PA01 *pilA* mutant with the D1585 *pilA* gene, expression of the exogenous D1585 *pilA* gene in our 280 Δ*pilA* mutant permitted infection by DLP2 as well as DLP1, plaquing at 10^5^ PFU/mL and 10^8^ PFU/mL respectively (Figure 5b). The reverse complementation of D1585 Δ*pilA* with the 280 *pilA* also restores DLP1 and DLP2 infection to near wildtype levels, with DLP2 infecting more efficiently (Figure 4, Table 3). Cross-genera complementation of 280 Δ*pilA* with the *P. aeruginosa* PA01 *pilA* gene did not restore infection by either DLP1 or DLP2. This is contrary to the reverse complementation of PA01 *pilA* mutants with the 280 *pilA* gene that shows partial infection by DLP2 as well as DLP1 (Figure 1, Table 3). These observations suggest that the *P. aeruginosa* PA01 PilA subunit does not assemble proficiently with the *S. maltophilia* 280 type IV pili machinery, whereas the more closely related D1585 PilA subunit can be assembled correctly to allow pili function and phage infection. The amino acid sequence identity between 280 and PA01 PilA subunits is lower than 280 and D1585 PilA, sharing 48% and 67% sequence identity respectively. Additionally, the twitching motility zone of 280 Δ*pilA* carrying pPA01pilA is reduced by 80% relative to wildtype 280, compared to complementation with the D1585 or endogenous *pilA* gene restoring twitching motility to 52% and 29% of wildtype respectively (Figure 5a).

While inefficient pilin assembly in foreign backgrounds may explain changes in phage susceptibility, it is also possible that *S. maltophilia* 280 modifies its surface pili to become unrecognizable by some bacteriophages, such as DLP1. Studies of pilus-specific phage in *P. aeruginosa* have revealed that surface modification of pili via glycosylation can protect the bacteria from phage infection by masking potential binding sites, without creating disadvantageous phenotypes through changes to pilin sequence [59]. While this modification protects *P. aeruginosa* from infection by most phages, some phages such as DMS3 have developed the ability to bind glycosylated pili and bypass this bacterial defense mechanism [59]. If *S. maltophilia* strain 280 has a modification system for its pili, this modification may mask DLP1′s binding site by steric hindrance; however, expressing the 280 *pilA* gene in a PA01 or D1585 background that lacks this modification system allows DLP1 to recognize a new motif for host recognition, resulting in more efficient infection than expression in 280 (Table 3).

Tail structures play an essential role in host cell recognition and penetration of the bacterial cell wall structure. Contractile tail *Myoviridae* phages typically possess tail fiber proteins to help stabilize the tail on the cell surface, whereas non-contractile tail *Siphoviridae* phages do not necessarily possess tail fibers [60]. One such fiberless phage is J-1, a temperate siphovirus isolated from an abnormal fermentation with *Lactobacillus casei*, which was noted for having no tail fibers [61]. Presumably, tail fibers are unimportant where the phage binding site is of limited size. Alternatively, head or tail tube ligands may provide additional attachment stabilization for the phage [62]. In the case of DLP1 and DLP2, initial binding to the type IV pili may obviate the need for tail fibers, as the pilin binding site is restrictively small. Instead, DLP1 and DLP2 binding appears to rely solely upon baseplate attachment to the pilin subunit, and genome ejection putatively only occurs after pilus retraction to the cell surface, making tail fibers unnecessary. Related *Siphoviridae* phages, all lacking the presence of known or annotated tail fiber genes, and some of which that have been determined to also bind type IV pili, possess similar central tail hub or major baseplate proteins carrying the pfam 13550 Phage-tail_3 domain (Table 4, Supplementary Figure S1 and Table S3). Our bioinformatic analysis suggests phages without encoded tail fibers, and with baseplate proteins possessing the Phage-tail_3 domain closely related to those of phages DLP1 and DLP2, use pili as a primary receptor to gain access to the host cell. Further experimental testing of this hypothesis is currently in progress in our laboratory.

The type IV pilus is a common receptor for many *P. aeruginosa* specific phages, including PO4 [63], F116 [64], DMS3 [65], MP22 [66], and MPK7 [67]; however this study is the first to identify the type IV pilus as the surface receptor for phages that infect *S. maltophilia*. The type IV pilus is a well characterized virulence factor in many bacteria, including *P. aeruginosa* and *N. gonorrhoeae*, involved in surface motility, biofilm formation, and adherence to mammalian cells and surfaces [44]. The results presented identify the type IV pilus as the primary receptor for both DLP1 and DLP2, with implications for phage therapy. Several studies have shown the ability of bacteriophages to increase bacterial virulence through moron genes encoded by the phage; however phages may also provide a selective pressure against bacteria expressing specific virulence factors [16]. Although bacteria may become resistant to phages through modification of phage receptors, when the phage receptor is a virulence factor such as lipopolysaccharide or type IV pili, this mutation provides resistance at the cost of lowered virulence and reduced fitness compared to non-resistant cells [16]. Therapy targeting bacterial virulence factors has been termed an “anti-virulence strategy” [68] and such a strategy using an antibiotic in combination with a phage targeting a *P. aeruginosa* efflux pump responsible for antibiotic resistance has been used successfully to treat a patient’s life-threatening aortic infection [15,69]. Therefore, the application of “anti-virulence” phages such as DLP1 and DLP2 may prove to be an effective therapy for clearing *S. maltophilia* and *P. aeruginosa* infections, while potentially reducing the virulence of resistant mutants that may arise.

## Figures and Tables

**Table 1:** List of bacterial strains, phages and plasmids used in this study.

Bacterial Strain	Genotype or Description	Source
*P. aeruginosa* PA01	Wildtype host for DLP1	[25]
*S. maltophilia* D1585	Wildtype host for DLP1 and DLP2	CBCCRRR *
D1585 Δ*pilA*	Clean deletion of *pilA* in D1585	This study
*S. maltophilia* 280	Wildtype host for DLP2	PLPHN/AHS **
280 Δ*pilA*	Clean deletion of *pilA* in 280	This study
*E. coli* S17-1	Conjugative donor strain	[26]
*E. coli* DH5α	Host for plasmid cloning	[27]
Phage		
DLP1	Lytic phage	Accession: KR537872.1 [17]
DLP2	Lytic phage	Accession: KR537871.1 [17]
φKZ	Lytic *Pseudomonas* phage	Accession: NC_004629.1 [23,28]
Plasmids		
pBBR1MCS	Broad-host range cloning vector, Cm^R^	[29]
pD1585pilA	pBBR1MCS carrying D1585 *pilA*, Cm^R^	This study
pPA01pilA	pBBR1MCS carrying PA01 *pilA*, Cm^R^	This study
p280pilA	pBBR1MCS carrying 280 *pilA*, Gm^R^	This study
pD1585pilE	pBBR1MCS carrying D1585 *pilE*, Cm^R^	This study
pPA01pilE	pBBR1MCS carrying PA01 *pilE*, Cm^R^	This study
pUCP22	Broad-host range cloning vector, Gm^R^	[30]
pUCP(D1585pilA)	pUCP22 carrying D1585 *pilA*, Gm^R^	This study
pUCP(PA01pilA)	pUCP22 carrying PA01 *pilA*, Gm^R^	This study
pUCP(280pilA)	pUCP22 carrying 280 *pilA*, Gm^R^	This study
pUCP(D1585pilE)	pUCP22 carrying D1585 *pilE*, Gm^R^	This study
pUCP(PA01pilE)	pUCP22 carrying PA01 *pilE*, Gm^R^	This study
pEX18Tc	Tc^R^, *oriT*, *sacB*, gene replacement vector	[31]
pD1585ΔpilA	pEX18Tc, 2 kb ΔpilA D1585 region	This study
p280ΔpilA	pEX18Tc, 2 kb ΔpilA 280 region	This study

* Canadian *Burkholderia cepacia* complex Research and Referral Repository; ** Provincial Laboratory for Public Health—North, Alberta Health Services.

**Table 2:** *P. aeruginosa* PA01 genes involved in type IV pilus biogenesis and DLP1 phage infection identified by a transposon mutant library screen.

Number of Mutants	Gene Affected	Function	DLP1 Lysis	Source
2	*pilA*	Major pilin subunit	−	[21]
4	*pilB*	Cytoplasmic ATPase/pilin polymerase	−	[20]
3	*pilE*	Minor pilin subunit	−	[20]
1	*pilF*	Outer membrane pilotin; controls secretin localization	−	[21]
1	*pilJ*	Involved in pilus assembly	−	[20]
1	*pilN*	Inner membrane assembly protein	−	[21]
2	*pilQ*	Secretin monomer; forms outer membrane pore	−	[21]
1	*pilR*	Cytoplasmic response regulator of two-component system; regulates PilA expression	−	[20]
4	*pilS*	Inner membrane histidine kinase of two component system; regulates PilA expression	−	[20]
2	*pilT*	Cytoplasmic ATPase; pilin depolymerase	−	[20]
1	*pilU*	Cytoplasmic ATPase; regulation of pilus retraction	+	[21]
2	*pilV*	Minor pilin subunit	−	[20]
4	*pilY1*	Possible adhesin; regulates pilus retraction	−	[20]
3	*fimV*	Inner membrane protein; aids in secretin assembly	+/−	[20]
1	*algR*	Regulates expression of minor pilin operon	+/−	[20]
1	*PA2806*	Conserved hypothetical protein	−	[20]

Strain characteristics: +, phage sensitivity; −, phage resistance; +/−, DLP1 low efficiency of plating.

**Table 3:** Summary of DLP1 and DLP2 phage susceptibility of cross complemented *pilA* mutants.

A. Strain + DLP1	Pilin Complement		
	pPA01*pilA*	pD1585*pilA*	p280*pilA*
***P. aeruginosa* PA01 *pilA^−^***	10^9^	10^8^	10^9^
***S. maltophilia* D1585** **Δ*pilA***	10^7^	10^3^	10^5^
***S. maltophilia* 280** **Δ*pilA***	-	10^8^	-
**B. Strain + DLP2**	**pPA01*pilA***	**pD1585*pilA***	**p280*pilA***
***P. aeruginosa* PA01 *pilA^−^***	-	10^7^	10^8^
***S. maltophilia* D1585** **Δ*pilA***	10^9^	10^3^	10^3^
***S. maltophilia* 280** **Δ*pilA***	-	10^5^	10^7^

Darker shading indicates increased susceptibility to phages: ◻ no infection, 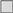 clearing at 10^9^, 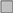 plaquing at 10^9^, 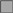 clearing at 10^8^, 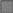 plaquing at 10^7^, 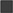 plaquing at 10^5^, ◼ plaquing at 10^3^.

**Table 4:** Amino acid sequence comparison of DLP1 and DLP2 phage central tail hub proteins containing the Pfam13550 Phage-tail_3 domain of *Siphoviridae* phages.

Bacteriophage	Accession	% Homology to DLP1 Pfam13550	% Homology to DLP2 Pfam13550
*Stenotrophomonas* phage DLP1 ^a^	AKI28788.1	-	98.3
*Pseudomonas* phage 73	YP_001293432	99.5	98.5
*Pseudomonas* phage vB_PaeS_C1	AVJ48095	98.8	99.3
*Pseudomonas* phage vB_Pae-Kakheti25	YP_006299890	98.5	99.1
*Pseudomonas* phage vB_Pae_PS9N	AIW01689	98.4	98.4
*Stenotrophomonas* phage DLP2 ^a^	AKI28730.1	98.3	-
*Pseudomonas* phage vB_PaeS_SCH_Ab26	YP_009044360	97.9	97.4
*Pseudomonas* phage PaMx42	YP_009205621	69.3	69.7
*Burkholderia* phage KL1	YP_006560777	46.8	46.8
*Xylella* phage Sano ^a,b^	AHB12068	29.5	29.4
*Xylella* phage Salvo ^a,b^	AHB12243	29	28.8

^a^ Experimentally confirmed as pili-binding phages. ^b^ One tail fiber gene annotated, but no tail fiber hits using nucleotide sequence for CD-search against database CDD v3.16–50369 PSSMs with the expected E-value threshold of 0.01, and composition-based statistics adjustment checked.

## Supplementary Materials

Supplementary materials can be found at http://www.mdpi.com/1999-4915/10/6/338/s1.
