# Supplementary material for: Identification and Characterization of Type IV Pili as the Cellular Receptor of Broad Host Range Stenotrophomonas maltophilia Bacteriophages DLP1 and DLP2
Source: Viruses. 2018 Jun 20;10(6):338. doi: 10.3390/v10060338 (PMC6024842; doi:10.3390/v10060338)
Supplement: Supplementary file 1 [file viruses-10-00338-s001.pdf]

Supplementary Table 1: Primers used in this study

Primer name	Sequence (5′ – 3′)	Function
Sm*pilA*upF	GTATGTCGACGCCAATCGCCCCTATGCTGG	Anneals 1,047 to 1,028 bp upstream to the D1585 *pilA* start codon, *SalI* site underlined
Sm*pilA*upR-OE	**ATGGGATTAGCAGCC**AGAGCACAGGATCGG TCTGG	The 20 nucleotides at the 3' end anneal to the seven codons following the D1585 *pilA* start codon and the first 15 nucleotides in bold overlap with the 5' end of the downstream fragment
Sm*pilA*downF-OE	**CCGATCCTGTGCTCT**GGCTGCTAATCCCATC TGGA	The first 15 nucleotides in bold overlap with the 3' end of the upstream fragment, the last 20 nucleotides at the 3' end anneal to the last three codons in D1585 *pilA* and 11 bp following the stop codon
Sm*pilA*downR	CCTCAAGCTTCCCCAACCACCTTGTTCTGC	Anneals 902 to 921 bp downstream to the D1585 *pilA* stop codon, *HindIII* site underlined
280*pilA*upR	GCCCAAGCTTCATGTTCACGATCATCTGGG	Anneals 1,046 to 1,027 bp upstream to the 280 *pilA* start codon on the reverse strand. *HindIII* site underlined.
280*pilA*upF-OE	**GGCGTACTTCTTCAG**CATTTTGGTACATCCC CAAG	The 20 nucleotides at the 3' end anneal to the 280 *pilA* start codon and 17 bp upstream, and the first 15 nucleotides in bold overlap with the 5' end of the downstream fragment.
280*pilA*downR-OE	**GGATGTACCAAAATG**CTGAAGAAGTACGCC CCGAC	The first 15 nucleotides in bold overlap with the 3' end of the upstream fragment, and the last 20 nucleotides at the 3' end anneal to seven out of 12 codons upstream of the 280 *pilA* stop codon
280*pilA*downF	GGCAGTCGACGGAACTTGATCTCGTCCAGC	Anneals 1,082 to 1,063 bp downstream of the 280 *pilA* stop codon. *SalI* site underlined.
Pa*pilA*F	GCGTGTCGACCCAGTTTCCTTGATCGTGGC	Anneals upstream of PA01 *pilA* gene. *SalI* site underlined.
Pa*pilA*R	GCCGAAGCTTGAGGAACCCAATCACAACGG	Anneals downstream of PA01 *pilA* gene. *HindIII* site underlined.
Pa*pilE*F	CCGAGGATCCGATCGAGAAAGAACAGCCCC	Anneals upstream of the PA01 *pilE* gene. *BamHI* site underlined.
Pa*pilE*R	GCGGAAGCTTGCGGGAGGAGAACATTACCT	Anneals downstream of the PA01 *pilE* gene. *HindIII* site underlined.
Sm*pilA*F	CCAAGTCGACCCATCCGTGAAATAGCTGCC	Anneals upstream of D1585 *pilA* start codon. *SalI* site underlined.
Sm*pilA*R	CGCCAAGCTTACGAGCCGACAAAAGAAAGGC	Anneals downstream of D1585 *pilA* stop codon. *HindIII* site underlined.
Sm*pilE*F	GTCTGTCGACCAGTAACCCCAGTGCGAGGA	Anneals upstream of the D1585 *pilE* gene. *SalI* site underlined.
Sm*pilE*R	GCCCAAGCTTCTAACCGGCTGAGCTATTCG	Anneals downstream of the D1585 *pilE* gene. *HindIII* site underlined.
280*pilA*F	GCAAGTCGACCAGACCGATCCTGTGCTCTG	Anneals upstream of the 280 *pilA* gene. *SalI* site underlined.
280*pilA*R	GACCAAGCTTCCCCTAGTTCGCTTCATGGC	Anneals downstream of the 280 *pilA* gene. *HindIII* site underlined.

Supplementary Table 2: Characteristics of *P. aeruginosa* PA01 transposon mutants

Mutant ID/ Strain Name	Gene affected	Transposon	Genome Insertion Position	DLP1 lysis	Source
PW8621	*pilA*	*lacZ-hah*	5069310	-	[21]
PW8622	*pilA*	*phoA-hah*	5069368	-	[21]
PA01_lux_18_G2	*pilB*	*mini-Tn5-luxCDABE*	5069913	-	[20]
PA01_lux_50_H10	*pilB*	*mini-Tn5-luxCDABE*	5071244	-	[20]
PA01_lux_67_D1	*pilB*	*mini-Tn5-luxCDABE*	5070860	-	[20]
PA01_lux_97_B10	*pilB*	*mini-Tn5-luxCDABE*	5070077	-	[20]
PA01_lux_38_F5	*pilE*	*mini-Tn5-luxCDABE*	5104853	-	[20]
PA01_lux_41_C7	*pilE*	*mini-Tn5-luxCDABE*	5104839	-	[20]
PA01_lux_50_D5	*pilE*	*mini-Tn5-luxCDABE*	5104869	-	[20]
PW7438	*pilF*	*phoA-hah*	4264656	-	[21]
PA01_lux_44_E9	*pilJ*	*mini-Tn5-luxCDABE*	452821	-	[20]
PW9471	*pilN*	*phoA-hah*	5679378	-	[21]
PW9465	*pilQ*	*phoA-hah*	5676840	-	[21]
PW9466	*pilQ*	*phoA-hah*	5676900	-	[21]
PA01_lux_80_E5	*pilR*	*mini-Tn5-luxCDABE*	5096068	-	[20]
PA01_lux_18_G4	*pilS*	*mini-Tn5-luxCDABE*	5094527	-	[20]
PA01_lux_42_D11	*pilS*	*mini-Tn5-luxCDABE*	5094527	-	[20]
PA01_lux_53_B6	*pilS*	*mini-Tn5-luxCDABE*	5094527	-	[20]
PA01_lux_80_C7	*pilS*	*mini-Tn5-luxCDABE*	5094527	-	[20]
PA01_lux_32_G12	*pilT*	*mini-Tn5-luxCDABE*	436863	-	[20]
PA01_lux_46_D4	*pilT*	*mini-Tn5-luxCDABE*	436504	-	[20]
PW1730	*pilU*	*lacZ-hah*	438793	+	[21]
PA01_lux_19_D2	*pilV*	*mini-Tn5-luxCDABE*	5098940	-	[20]
PA01_lux_73_C10	*pilV*	*mini-Tn5-luxCDABE*	5099241	-	[20]
PA01_lux_20_D4	*pilY1*	*mini-Tn5-luxCDABE*	5101178	-	[20]
PA01_lux_51_H7	*pilY1*	*mini-Tn5-luxCDABE*	5102516	-	[20]
PA01_lux_82_C12	*pilY1*	*mini-Tn5-luxCDABE*	5102516	-	[20]
PA01_lux_97_G2	*pilY1*	*mini-Tn5-luxCDABE*	5100755	-	[20]
PA01_lux_67_E3	*fimV*	*mini-Tn5-luxCDABE*	3496099	+/-	[20]
PA01_lux_20_D1	*fimV*	*mini-Tn5-luxCDABE*	3497859	+/-	[20]
PA01_lux_21_F1	*fimV*	*mini-Tn5-luxCDABE*	3496114	+/-	[20]
PA01_lux_97_D2	*algR*	*mini-Tn5-luxCDABE*	5923165	+/-	[20]
PA01_lux_50_H9	*PA2806*	*mini-Tn5-luxCDABE*	3160973	-	[20]

More information on strains is available at http://pseudomutant.pseudomonas.com for *mini-Tn5-luxCDABE* mutants and http://www.gs.washington.edu/labs/manoil/libraryindex.htm for *lacZ-hah* and *phoA-hah* mutants.

Supplementary Table 3: Comparison of the presence of Phage-tail_3 domain and tail fiber domains of experimentally confirmed pili-receptor phages.

Bacteriophage	Accession	Morphology	Phage-tail_3 domain	Tail fiber domains
Stenotrophomonas phage DLP1	KR537872.1	*Siphoviridae*	Yes	-
Stenotrophomonas phage DLP2	KR537871.1	*Siphoviridae*	Yes	-
Stenotrophomonas phage DLP4	MG018224.1	*Siphoviridae*	Yes	Yes (1x)
Pseudomonas phage D3112	NC_005178	*Siphoviridae*	Yes	-
Pseudomonas phage MP22	NC_009818	*Siphoviridae*	Yes	-
Pseudomonas phage B3	NC_006548	*Siphoviridae*	Yes	-
Pseudomonas phage JBD26	JN811560	*Siphoviridae*	Yes	-
Pseudomonas phage JBD68	KY707339	*Siphoviridae*	-	-
Pseudomonas phage MP29	EU272036.1	*Siphoviridae*	Yes	-
Pseudomonas phage MP42	JQ762257.1	*Siphoviridae*	Yes	-
Pseudomonas phage JBD69	KU199708.1	*Siphoviridae*	Yes	-
Pseudomonas phage JBD93	NC_030918.1	*Siphoviridae*	Yes	-
Pseudomonas phage JBD88a	NC_020200.1	*Siphoviridae*	-	-
Pseudomonas phage JBD5	NC_020202.1	*Siphoviridae*	Yes	-
Pseudomonas phage JBD30	NC_020198.1	*Siphoviridae*	Yes	-
Xylella phage Paz	KF626666	*Siphoviridae*	-	Yes (1x)[a]
Xylella phage Prado	KF626667	*Siphoviridae*	-	Yes (1x)[a]
Xylella phage Salvo	KF626668	*Siphoviridae*	Yes	-[b]
Xylella phage Sano	KF626665	*Siphoviridae*	Yes	-[b]
Caulobacter phage phiCbK	JX100813	*Siphoviridae*	Yes	-
Pseudomonas phage phiKMV	AJ505558	*Podoviridae*	-	Yes (1x)[c]
Pseudomonas phage MPK7	JX501340	*Podoviridae*	-	Yes (2x)
Pseudomonas phage LUZ19	NC_010326	*Podoviridae*	-	Yes (3x)
Pseudomonas phage F116	NC_006552	*Podoviridae*	-	-
Pseudomonas phage Pf-10	NC_027292	*Podoviridae*	-	Yes (1x)
Pseudomonas phage JBD23	KM389462.1	N/A[d]	Yes	-

a. Three tail fiber genes annotated, but only one tail fiber hit on CD-search; therefore, only counted as 1x tail fiber domains.

b. One tail fiber gene annotated, but no tail fiber hits on CD-search, therefore not counting as tail fiber domains.

c. Two tail fiber proteins annotated, but only one hit on CD-search; therefore, only counting as 1x tail fiber domains.

d. Worked with phage experimentally in a lab (Bondy-Delomy *et. al.,* 2016. Prophages mediate defense against phage infection through diverse mechanisms. ISME Journal (10); 2854–2866) and sequenced, but no annotations or TEM images.

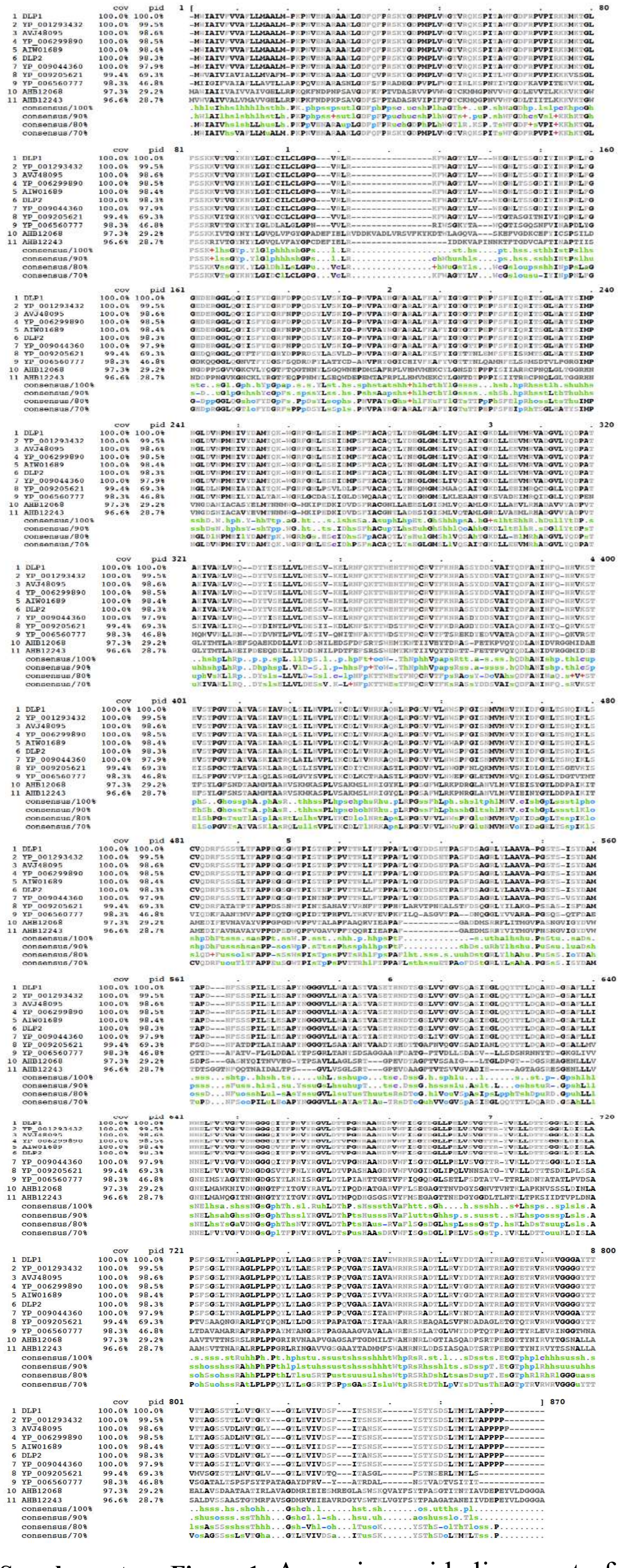

Supplementary Figure 1. An amino acid alignment of the DLP1 and DLP2 baseplate or central tail hub proteins with their ten nearest neighbours. Black lettered amino amino acids are identical; lighter shaded amino acids have less homology. Consensus cutoff levels and amino

acids are shown below the grouping. The alignment was performed using EMBL-EBI Multiple Sequence Alignment Tool and visually presented using M-view
